# Xenon Protects against Blast-Induced Traumatic Brain Injury in an *In Vitro* Model

**DOI:** 10.1089/neu.2017.5360

**Published:** 2018-04-15

**Authors:** Rita Campos-Pires, Mariia Koziakova, Amina Yonis, Ashni Pau, Warren Macdonald, Katie Harris, Christopher J. Edge, Nicholas P. Franks, Peter F. Mahoney, Robert Dickinson

**Affiliations:** ^1^ Anaesthetics, Pain Medicine and Intensive Care Section, Department of Surgery and Cancer, Imperial College London, London, United Kingdom.; ^2^ Royal British Legion Centre for Blast Injury Studies, Department of Bioengineering, Imperial College London, London, United Kingdom.; ^3^ Department of Bioengineering, Imperial College London, London, United Kingdom.; ^4^ Department of Life Sciences, Imperial College London, London, United Kingdom.; ^5^ Department of Anaesthetics, Royal Berkshire Hospital NHS Foundation Trust, Reading, United Kingdom.; ^6^ Royal Centre for Defence Medicine, Medical Directorate Joint Force Command, ICT Centre, Birmingham, United Kingdom.

**Keywords:** blast-induced neurotrauma, blast traumatic brain injury, neuroprotection, primary blast injury, TBI, xenon

## Abstract

The aim of this study was to evaluate the neuroprotective efficacy of the inert gas xenon as a treatment for patients with blast-induced traumatic brain injury in an *in vitro* laboratory model. We developed a novel blast traumatic brain injury model using C57BL/6N mouse organotypic hippocampal brain-slice cultures exposed to a single shockwave, with the resulting injury quantified using propidium iodide fluorescence. A shock tube blast generator was used to simulate open field explosive blast shockwaves, modeled by the Friedlander waveform. Exposure to blast shockwave resulted in significant (*p* < 0.01) injury that increased with peak-overpressure and impulse of the shockwave, and which exhibited a secondary injury development up to 72 h after trauma. Blast-induced propidium iodide fluorescence overlapped with cleaved caspase-3 immunofluorescence, indicating that shock-wave–induced cell death involves apoptosis. Xenon (50% atm) applied 1 h after blast exposure reduced injury 24 h (*p* < 0.01), 48 h (*p* < 0.05), and 72 h (*p* < 0.001) later, compared with untreated control injury. Xenon-treated injured slices were not significantly different from uninjured sham slices at 24 h and 72 h. We demonstrate for the first time that xenon treatment after blast traumatic brain injury reduces initial injury and prevents subsequent injury development *in vitro*. Our findings support the idea that xenon may be a potential first-line treatment for those with blast-induced traumatic brain injury.

## Introduction

Traumatic brain injury (TBI) is a leading cause of death and disability in both military and civilian populations,^1–3^ but there are, as yet, no effective treatments targeting injury development. Blast-TBI has been called a “signature injury” of recent military operations in Iraq and Afghanistan.^2,3^ While blunt and penetrating TBI injury have been studied extensively, blast-TBI is much less well understood but is being recognized now as having a unique pathophysiology.^4^ The prevalence of blast-injury in recent returning veteran populations^5,6^ has prompted research into blast-TBI. Studies in rodents, swine, and other mammals have shown that exposure to blast-overpressure can result in a variety of behavioral impairments and neuropathological abnormalities.^7–9^

Despite the increasing research focus on blast-TBI pathophysiology, there are no clinically proven treatments to prevent or limit ongoing brain injury after blast-TBI. In addition, to date there have been few pre-clinical studies evaluating potential treatments or reporting improved outcomes after blast-TBI.^8,10,11^ There is an urgent need for treatments aimed at mitigating the neurological and cognitive deficits caused by blast-TBI and promoting a more rapid and complete recovery.

Xenon is a nonflammable inert gas that has been used as a general anesthetic since the 1950s.^12,13^ Xenon has been shown to be neuroprotective in a variety of *in vitro* and *in vivo* models of ischemia and stroke.^14–23^ Xenon is a pleiotropic drug known to act via a number of targets implicated in secondary injury development including inhibition of N-methyl-D-aspartate receptors,^24–26^ activation of potassium channels,^27,28^ and anti-apoptotic action.^29^ Xenon has a number of unique advantages including not being metabolized and rapidly crossing the blood–brain barrier facilitating a rapid onset and offset of action, within minutes. A recent clinical trial of xenon for ischemic brain injury in patients with out-of-hospital cardiac arrest showed that xenon administered within 6 h after injury reduces cerebral white matter damage.^30^

We have shown recently that xenon mitigates brain injury progression and improves long-term outcome in blunt-TBI in rodents.^31^ Here we describe a novel *in vitro* model of blast-TBI, which we utilize to test the hypothesis that xenon treatment can prevent or limit brain injury after blast-wave exposure.

## Methods

Experiments complied with the United Kingdom (UK) Animals Scientific Procedures Act (1986) and were approved by the Animal Welfare and Ethical Review Body of Imperial College London. Unless otherwise stated, reagents were purchased from Sigma Aldrich (Dorset, UK).

### The shock tube blast generator

**FIG. 1.**
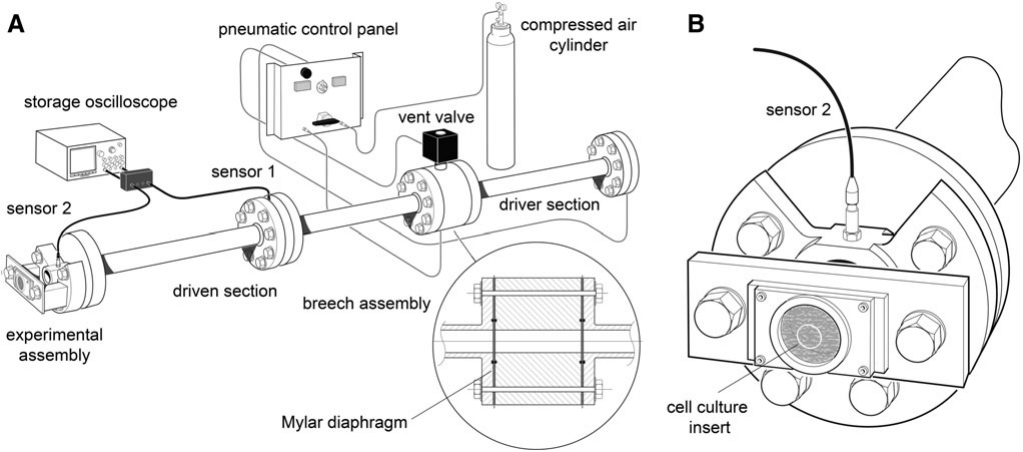
**(A**) Schematic drawing of the shock tube used for these studies. The driver section is filled with compressed air via a control panel with control valves and pressure gauges allowing separate control of the pressure in the breech and the driver tube. A solenoid-controlled vent valve allows the pressure in the breech to be rapidly vented, triggering diaphragm rupture in the double-breech configuration. The breech (inset) can be used in single-diaphragm or double-diaphragm configuration. The driven section (left of the breech) has two ultrafast rise-time dynamic pressure transducers, mounted radially, halfway along (sensor 1) and at the distal end (sensor 2). These sensors are connected to a high bandwidth digital storage oscilloscope. (**B**) Detail of the shock tube assembly used in these *in vitro* experiments. The Millicell culture insert (small grey circle) with organotypic slices is inside a polythene bag containing experimental medium. The polythene bag is clamped all around a circular aperture in front of the shock-tube distal flange.

A shock tube was used to generate controlled overpressure waves that model real-life free-field explosions with a Friedlander-type waveform.^32^ The shock tube (Fig. 1A) is a 3.8 m long horizontal stainless steel tube, with three 1.22 m long sections, with internal diameter of 0.059 m and external diameter of 0.073 m. The first section, 1.22 m long, is the high pressure driver section, separated from the low pressure (1 atm) section, 2.44 m long, by a double breech assembly, 0.08 m long. The double breech allows one or two Mylar^®^ polyester diaphragms (RS Components, Northants, UK) to be clamped between the high pressure driver section and the driven section with gas tight seals provided by nitrile O-rings (Fig. 1A inset). The effective driver volume can be adjusted by including one or more cylindrical polyethylene “blanking” sections inside the driver section.

For these *in vitro* experiments, we used a 15% driver volume in the single and double diaphragm configuration to generate shockwaves with peak overpressures of 55 kPa and 50 kPa, respectively. The driver section was pressurized with compressed air (BOC, Guildford, UK). Burst pressure is determined by the diaphragm material and thickness and in the double diaphragm configuration is triggered by venting the breech section through a fast-acting servo-valve. Two ultrafast piezoelectric high dynamic pressure transducers (2300 V1, Dytran Instruments, Chatsworth, CA) were mounted radially on the shock tube. Sensor 1, mounted in the middle of the driven section, was used for triggering data acquisition; sensor 2, mounted at the end of the driven section, was used for measurement of shockwave peak overpressure and duration (Fig. 1). The pressure transducers were connected to a current source power unit (model 4103C, Dytran Instruments) and output signals recorded on a high bandwidth oscilloscope (model DPO4104B, Tektronix Inc, Beaverton, OR).

Shockwave data were acquired at a sampling rate of 50 MHz over 20 msec (1 × 10^6^ samples/wave). Voltage signals were saved on digital storage media and analyzed offline on a computer using MATLAB software (Release 2015a, MathWorks, Natick, MA) and the appropriate calibration factor for each pressure transducer provided by the manufacturer.

### *In vitro* slice cultures

Organotypic hippocampal slice cultures (OHSCs) were prepared from postnatal day five to seven C57BL/6N mouse pups under aseptic conditions using the interface method described by Stoppini and associates^33^ with some modifications.^34–36^ Slices on tissue culture inserts (Millicell-CM, Millipore, Carrigtwohill, Ireland) were cultured at 37°C with 5% CO_2_ in air in a humidified incubator (BB6220, Heraeus, Germany) for 12–14 days. The growth medium was changed on the first day of culture and every two to three days thereafter.

### Preparation of OHSCs for *in vitro* blast-TBI

After 12–14 days in culture, the tissue culture inserts were transferred to six-well culture plates (Nunc, Roskilde, Denmark) containing pre-warmed (37°C) serum-free “experimental medium” with propidium iodide (PI) (75% minimum essential medium Eagle; 25% Hank balanced salt solution; 5 mg/mL d-glucose; 2 mM l-glutamine; 1% antibiotic-antimycotic solution; 10 mM HEPES; 4.5 μM PI; pH titrated to 7.2). At 1 h after transfer to experimental medium, the slices were imaged (see *Quantifying cell injury*) to assess baseline slice health before injury and to ensure that all slices were equivalent at the start of the procedure (and before allocating to experimental groups). A small number of slices exhibited areas of dense staining at this stage (likely because of mechanical damage occurring during slice preparation) and were excluded from further analysis.

Immediately after baseline imaging, individual tissue culture inserts were sealed carefully in sterile polyethylene sample bags (Twirl'em^®^ 3” × 5”, Fisher Scientific, Loughborough, UK) using aseptic conditions. Each bag was pre-filled with warmed (37°C) experimental medium that had been saturated by bubbling with 95%O_2_; 5%CO_2_ for 45 min. Gas bubbles were carefully excluded on sealing sample bags. We determined whether slice submersion affected slices and found no significant difference between slices that had been maintained without submersion and those submerged (data not shown).

### *In vitro* blast injury procedure

**FIG. 2.**
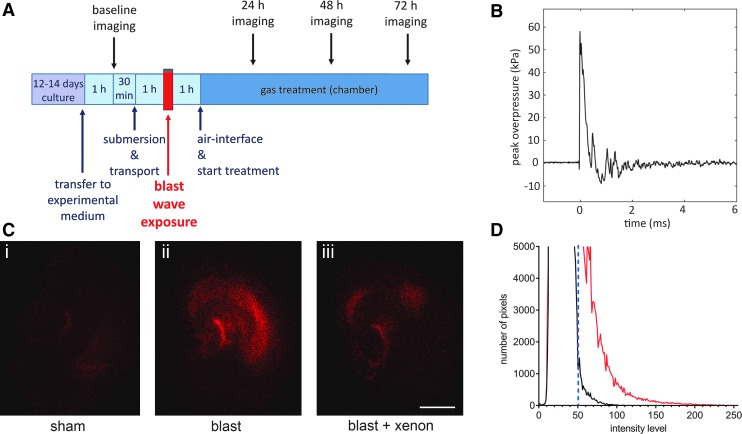
(**A**) Experimental timeline. (**B**) A representative shockwave obtained using single 23-μm Mylar^®^ diaphragm, peak overpressure 55 kPa, positive wave duration 0.4 msec, and impulse 10.3 kPa•msec. Shockwave data were obtained from a pressure transducer mounted radially on the distal flange of the driven section. (**C**) Images showing typical (**i**) uninjured sham slice, (**ii**) blast-TBI exposed slice, and (**iii**) xenon-treated blast-TBI slice, at 72 h after injury. The red propidium iodide fluorescence is used as a marker of dead or dying cells. Scale bar = 500 μm. (**D**) For each slice, an image intensity histogram was produced and injury quantified by counting the number of pixels above a threshold of 50 in the intensity histograms (dashed vertical line). The lines shown indicate the intensity histograms of images of a typical sham slice (black line) and a typical blast-injured slice (red line) at 72 h after the blast or sham procedure.

At 1 h after submersion of the tissue culture insert, each sample bag was assigned randomly to either blast or sham group and clamped symmetrically all round in a vertical position in front of the shock-tube outlet using a custom-made stainless steel assembly (Fig. 1B) with the inserts positioned perpendicular to the axis of the shock tube with the OHSCs facing the shock tube. The OHSCs in the blast groups then were exposed immediately to a single shockwave (Fig. 2B). After blast-injury or sham procedure, each sample bag was placed in a thermo-regulated box (37°C) before OHSCs were returned to air-liquid interface conditions.

Our objective was to develop a model that was sensitive to injury induced by a primary blast wave. Because of the sensitivity of our measurement of cell death, and in common with other *in vitro* brain injury models,^35,36^ there is a degree of injury in the sham slices. For this reason, we ensured that every experiment included a sham group, and we randomly allocated slices to sham and blast groups. We took care to ensure that sham slices were treated identically to the slices exposed to a shockwave (sealed in the sample bags and suspended on the stainless steel rig for an equivalent period), but the shock tube was not fired. The level of injury in the sham slices at 24 h and 48 h was similar to that observed in our *in vitro* blunt TBI model,^36^ but at 72 h was greater than the blunt TBI model, likely reflecting the greater slice manipulation in the blast model. The injury in the blast-exposed slices was significantly (*p* < 0.01) greater than sham slices at all time points after blast.

In some of the earlier experiments, to be able to distinguish between the different magnitudes of blast (50 kPa; 55 kPa) and to assess how injury developed after both blast intensities, we had larger group sizes (∼30–50 slices). In the xenon neuroprotection experiments, we chose the higher blast intensity (55 kPa), and in these experiments, smaller group sizes (∼12) were used for the injury and treatment groups; because we included a sham group per condition in every experiment, the sham group was larger (∼23).

### Experimental gas treatment

After the blast-TBI procedure, inserts were carefully removed from the sample bags inside a laminar flow tissue culture hood and transferred to six-well culture plates with pre-warmed (37°C) experimental medium. The six-well plate was transferred back to either the humidified incubator (37°C, 5% CO_2_ in air) or, for the gas treatment experiments, plates were randomly assigned to either xenon or control gas (helium) groups and transferred to one of two small custom-made hyperbaric chambers containing a high-speed fan for rapid gas mixing, housed in an incubator set at 37°C.^34,35^ The chamber (volume 0.925 L) was flushed with humidified control gas (20%O_2_; 75%N_2_; 5%CO_2_) for 5 min at 5 L/min.^36^ The chamber was then sealed and an additional 50% atm of helium or xenon was added (the total final pressure inside the chamber was 1.5 atm). The partial pressures of O_2_ and CO_2_ were fixed at 0.2 atm and 0.05 atm, respectively.

Six-well plates were removed from the chamber at 24 h, 48 h, and 72 h for imaging (Fig. 2A). For quantification of injury, slices in six well plates were allocated a well identifier A–F and a slice number (e.g., 1–4). Imaging was performed and data files stored with reference to these identifiers until the final analysis. After imaging, plates were transferred back to the chamber and the appropriate gas mixture was reestablished.

### Quantifying cell injury

The PI only enters cells with compromised cellular membranes and becomes fluorescent after binding to nucleic acids allowing quantification of cell injury.^37–39^ An epifluorescence microscope (Nikon Eclipse 80i, Kingston upon Thames, UK) with a low power objective (Nikon Plan UW 2 × magnification, NA 0.06) was used to visualize PI fluorescence (Fig. 2C). A digital camera (Micropublisher 3.3 RTC) and image capture software (QCapturePro, Qimaging Inc., Surrey, British Columbia, Canada), were used as described previously.^36^ Image intensity analysis of the red channel was performed using ImageJ software,^40^ with the distribution of intensities plotted as a histogram over 256 intensity levels. Slices under uninjured conditions exhibited a well-defined peak in the intensity distribution, which fell rapidly to zero. As a measure of injury caused by the shockwave exposure, the number of pixels above a threshold of 50 was integrated (Fig. 2D).^36^

### Immunofluorescence

At the end of the experiment, slices on Millicell inserts were washed with warmed (37°C) phosphate-buffered saline (PBS) followed by fixation for 1 h with 4% paraformaldehyde (ThermoFisher, Loughborough, UK) in PBS. Slices were detached carefully from inserts and mounted on glass slides. Slices were blocked for 90 min with 10% goat serum in PBS, incubated overnight at 4°C with cleaved caspase-3 primary antibody (9661S, Cell Signaling Technology, Leiden, Netherlands, dilution 1:200) followed by 1 h incubation at room temperature with Alexa Fluor^®^ 488 goat anti-rabbit secondary antibody (A-11008, Life Technologies, Paisley, UK, dilution 1:500), with 3 × washes with PBS 0.3% Triton X 100 between each step. Slices were imaged using a Nikon Eclipse 80i microscope with a × 100 objective (Nikon Plan Fluor, NA 1.3 oil, WD 0.20).

### Statistics

Results are expressed as mean ± standard error of the mean. Significance was assessed using a two-way repeated measures analysis of variance with Holm-Sidak *post hoc* test. Factor 1 was treatment (sham, xenon, control), and factor 2 was time before or after the injury (-1 h, 24 h, 48 h, and 72 h), where factor 1 was the repeated factor. Statistical tests were implemented using GraphPad Prism (GraphPad, La Jolla, CA).

## Results

### *In vitro* blast-TBI model

**FIG. 3.**
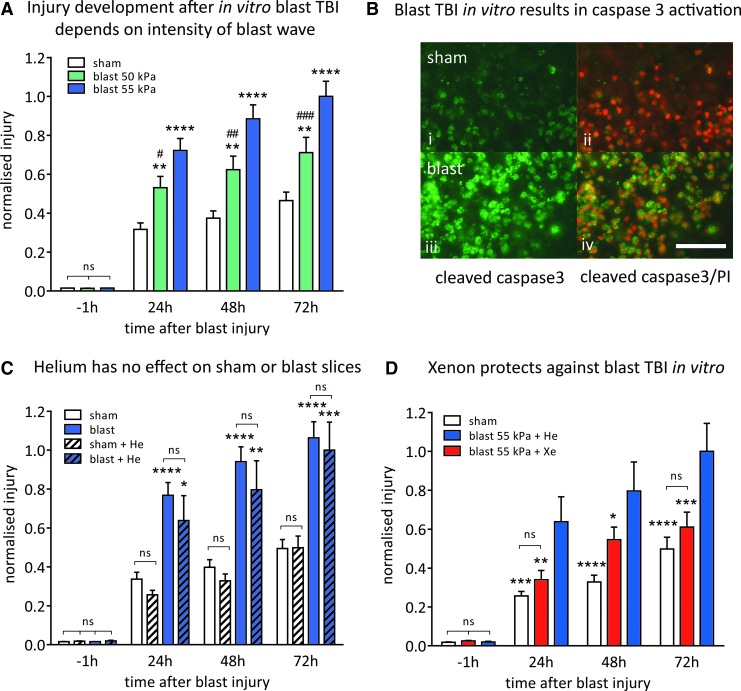
**A)** The magnitude of the developing injury is dependent on the intensity of the shockwave. Shockwaves with peak overpressures of 50 kPa (8.8 kPa•msec impulse) (green bars) and 55 kPa (10.3 kPa•msec impulse) (blue bars), resulted in a significant injury compared with uninjured sham-slices (white bars). Data have been normalized to control blast 55 kPa at 72 h after injury. Bars represent mean values and error bars are standard errors (*n* = 48, sham; *n* = 30, blast 50 kPa; *n* = 51, blast 55 kPa). **p < 0.01, ****p < 0.0001 compared with sham. #*p* < 0.05, ##*p* < 0.01; ###*p* < 0.01 compared with 55 kPa. **(B**) Blast-TBI results in activation of caspase-3 and cell death. (**i**) Uninjured sham stained for cleaved caspase-3 (green); (**ii**) overlay of cleaved caspase-3 (green) and propidium iodide (PI) (red) in uninjured sham slice; (**iii**) slice exposed to 55 kPa shockwave showing increase in cleaved caspase-3 staining (green); (**iv**) overlay of cleaved caspase-3 (green) and PI (red) in 55 kPa shockwave exposed slice showing co-localization of both makers. Scale bar = 50 μm. (**C**) Helium (50% atm) has no effect on the sham or 55 kPa blast-injured slices. Sham-treated slices are shown as white bars (no helium) or white hatched bars (helium), and blast-injured slices are shown as blue bars (no helium) or blue hatched bars (helium). Data have been normalized to control blast +50% atm helium at 72 h after injury. Bars represent mean values, and error bars are standard errors (*n* = 48, sham; *n* = 51, blast; *n* = 22, sham +50% atm helium; *n* = 12, blast +50% atm helium). *p < 0.05, **p < 0.01, ***p < 0.001, ****p < 0.0001 compared with the respective sham. (**D**) Xenon (50% atm) (red bars) prevents injury developing after exposure to 55 kPa shockwave. Xenon-treated blast-TBI slices (red bars) have reduced injury development compared with control slices treated with helium (blue bars). Xenon-treated blast-TBI slices were not significantly different from uninjured sham slices (white bars). Data have been normalized to control blast at 72 h after injury. Bars represent mean values, and error bars are standard errors (*n* = 22, sham; *n* = 11, blast +50% atm xenon; *n* = 12, blast +50% at helium). *p < 0.05, **p < 0.01, ***p < 0.001; ****p < 0.0001 compared with control blast. He, helium; Xe, xenon.

To determine the optimum level of traumatic injury for neuroprotection experiments, we determined how the peak overpressure (POP) of Friedlander waveform shockwaves (Fig. 2B) affected injury magnitude and development. The OHSCs exposed to blast-injury exhibited increased PI fluorescence, compared with identically treated sham slices (Fig. 2C i, ii). In all experiments, to ensure that slices allocated to each experimental group were the same at the start of the procedure, we measured the PI fluorescence 1 h before starting the blast injury protocol. The baseline fluorescence at t = −1 h was very low (typically 2% of control injury at 72 h) and was not significantly different between any of the groups (Fig. 3A,C,D). We quantified injury development as a function of shockwave peak overpressure, at 24 h, 48 h, and 72 h after injury (Fig. 3A).

Shockwaves with POPs of 50 kPa (duration 0.4 msec; impulse 8.8 kPa•msec) and 55 kPa (duration 0.4 msec; impulse 10.3 kPa•msec) resulted in injury that developed up to 72 h after blast exposure. Injury magnitude increased with shockwave intensity; the 55 kPa POP shockwave produced a significantly (*p* < 0.05) larger injury than the 50 kPa POP shockwave, at 24 h, 48 h, and 72 h after blast-injury (Fig. 3A). Shockwaves of 55 kPa POP were chosen as our standard injury because they produced a consistent and reproducible traumatic injury that developed significantly (*p* < 0.0001) over 72 h, compared with uninjured slices.

Blast-TBI–induced cell death involves apoptotic pathways as shown by an increase in cleaved caspase-3 after blast and strong colocalization with the PI staining (Fig. 3B). In contrast, in sham slices, not exposed to blast, while there was a low level of cell death indicated by PI staining, this was not associated with caspase activation (Fig. 3B).

### Lack of effect of helium

Because the xenon experiments would use 50% atm xenon, we determined whether pressure *per se* would affect the injury. The effect of 50% atm helium on sham-treated and blast-injured slices was investigated (Fig. 3C). Helium was chosen because we have shown in other *in vitro* brain injury models that helium at these low pressures does not affect OHSCs.^35,36^ Adding 50% atm helium had no significant effect (*p* > 0.87) on either the sham or blast-injured slices at any time point. Nevertheless, in all the experiments with xenon, we used 50% atm helium as the control.

### Neuroprotection by xenon

We evaluated the neuroprotective efficacy of treatment with 50% atm xenon starting 1 h after blast-TBI. Xenon had a protective effect against blast trauma at all time points (Fig. 3D). Injury in the xenon-treated slices was reduced by 47 ± 12% (*p* < 0.01) compared with the untreated injured slices 24 h after injury. In the xenon-treated group at 48 h after blast exposure, injury was reduced by 31 ± 7% (*p* < 0.05). At 72 h after injury, the injury was reduced by 39 ± 7% (*p* < 0.001) in the xenon-treated group. The blast-injured slices treated with xenon were not significantly different from the uninjured sham slices at 24 h and 72 h after injury.

## Discussion

We developed an *in vitro* model of blast-TBI using organotypic hippocampal slice cultures, based on a blunt-TBI model that we have used to investigate the neuroprotective efficacy and mechanism of action of a variety of inert gases including xenon.^36^ The *in vitro* blunt-TBI model was first described by Coburn and colleagues,^34^ who were the first to show xenon to be protective in that model. We modified the blunt-TBI model to include submersion of slices on tissue culture inserts inside polythene sample bags, allowing them to be exposed to shockwaves from a horizontal gas-driven shock tube maintaining aseptic conditions.

Our blast model has some similarities to *in vitro* blast-TBI models described by Miller and coworkers^41^ and Effgen and associates.^42^ In our *in vitro* blast-TBI model, we observed an injury that increased with the magnitude of the peak overpressure and which exhibited a progressive injury developing over the course of 72 h. This developing injury is similar to that observed in the *in vitro* blunt-TBI model^34,36^ and qualitatively similar to that observed in the blast-TBI models of Miller and coworkers^41^ and Effgen and associates.^42^ Our results are similar to those of Miller and coworkers^41^ who observed increases in PI fluorescence as early as 2 h after injury that developed up to 24 h, using shockwaves of 147 kPa POP, 10.4 kPa•msec impulse, and 278 kPa POP, 18.1 kPa•msec impulse.

There are some quantitative differences between our model and that of Effgen and colleagues.^42^ We observe blast-induced cell death at POP of 55 kPa (impulse 10.3 kPa•msec) that is lower than the injury threshold for cell death observed by Effgen of 424 kPa POP, 248 kPa•msec impulse.^43,44^ Interestingly, Vogel, Effgen, and colleagues^44^ observed changes in the electrophysiological properties of slices at impulse levels as low as ∼39 kPa•msec, which are closer to the impulse levels in our experiments.

In our model, we observe blast-induced cell death significantly greater than sham levels at time points as early as 24 h with development up to 72 h, whereas in the model of Effgen and coworkers,^42^ injury does not develop above sham level until 96 h after blast-injury. These differences may reflect the fact that our model used slices in a sterile bag while their model used a liquid filled receiver under a vertical shock tube, or the fact that we measured injury over the entire area of the slice while Effgen and coworkers^42^ used an approach based on region of interest, which may be less sensitive to a diffuse injury, such as the one caused by the exposure of OHSCs to a shockwave. We observed that PI fluorescence overlapped with cleaved caspase-3 immunostaining in blast-exposed slices but not sham slices, indicating that the blast-associated cell death involves activation of apoptotic pathways.

### Xenon neuroprotection

Treatment of the slices with 50% atm xenon, beginning 1 h after blast injury, caused a reduction in the degree of injury (cell death) at all time points tested, with xenon treatment reducing injury by between 31% and 47%. The degree of protection against blast injury that we observed with 50% xenon is similar to that observed in the *in vitro* blunt-TBI model.^34,36^ At the 24 h and 72 h time points, the xenon-treated injured slices were not significantly different from the uninjured sham slices, consistent with xenon preventing injury development after blast-TBI *in vitro*.

The finding that xenon prevents blast injury developing up to 72 h after trauma is novel and is relevant to the potential clinical use of xenon, because clinical TBI lesions may develop significantly in the first 24 h after injury.^45,46^ The degree of protection that we observed with xenon is notable given that in our *in vitro* experimental protocol, xenon treatment only begins 1 h after injury, to simulate a clinically relevant time interval before medical care arrives after the blast injury. This is consistent with the time window for xenon neuroprotection observed in *in vitro*^34^ and *in vivo*^31^ models of blunt TBI and is relevant for clinical translation. We observed the greatest effect of xenon treatment on injury development within the first 24 h, suggesting that treatment duration of 24 h or less may be effective. We have shown in an animal model of blunt TBI that xenon treatment for only three hours results in significant neuroprotection.^31^

### Limitations of the study

Organotypic slice cultures are more complex than simple dissociated cell cultures, retain a heterogeneous population of cell types whose synaptic connectivity mirrors that seen *in vivo,*^47–49^ and are a useful tool to evaluate efficacy and mechanism of novel neuroprotective treatments.^35,36^ They cannot replace animal models to investigate injury development and neuroprotection, however. The use of a shock tube to generate Friedlander waves allows the isolated brain tissue to be subject to mechanical loading of short duration (∼0.4 msec) modelling primary blast injury. Our *in vitro* model, however, cannot incorporate all of the other features of a real blast exposure *in vivo,* such as blunt injury (secondary blast), accelleration/deceleration (tertiary blast), or heat (quaternary blast). Nevertheless, *in vitro* models such as ours are a useful step in the translational process from bench to clinic.

## Conclusion

The current study describes a novel *in vitro* model of blast-TBI. We show that injury magnitude depends on the intensity of the shockwave and that exposure to blast initiates caspase-dependent cell death. We demonstrate for the first time that treatment with xenon, starting 1 h after trauma, significantly limits injury progression after blast-induced TBI *in vitro.* Xenon could be delivered easily by inhalation shortly after brain injury with relatively simple equipment. In addition to its potential for arresting injury development, xenon has an additional advantage of simultaneously providing analgesia.^50,51^ Our findings support the idea that xenon may be a potential treatment for those with blast-induced TBI, and further research in this area is merited.
